# Supplementary material for: The interactome of intact mitochondria by cross-linking mass spectrometry provides evidence for coexisting respiratory supercomplexes
Source: Mol Cell Proteomics. 2017 Dec 8;17(2):216–32. doi: 10.1074/mcp.RA117.000470 (PMC5795388; doi:10.1074/mcp.RA117.000470)
Supplement: Supplemental Data [file supp_RA117.000470_133922_0_supp_23978_fzffwf.zip › spectra_annotation/mito_DR_spectra_annotation/345-1-3-1-9-1.pdf]

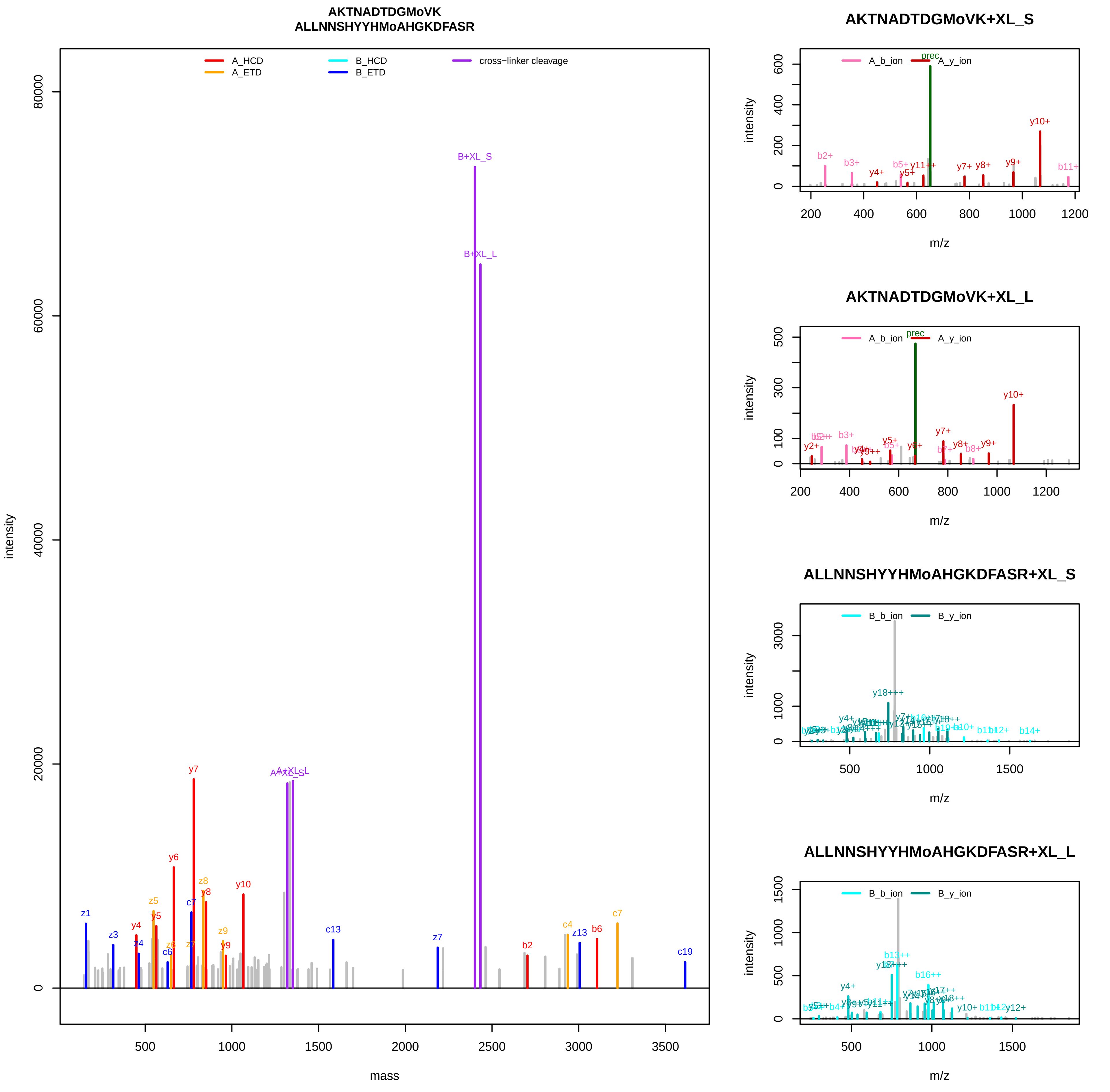
AKTNADTDGMoVK
ALLNNSHYYHMoAHGKDFASR
A_HCD
A_ETD
B_HCD
B_ETD
cross−linker cleavage
B+XL_S
B+XL_L
intensity
mass
80000
60000
40000
20000
0
500
1000
1500
2000
2500
3000
3500
z1
z3
y4
z4
z5
y5
c6
y6
y7
c7
z7
z8
y8
z9
y9
y10
c13
z7
b2
c4
z13
b6
c7
c19
AKTNADTDGMoVK+XL_S
A_b_ion
A_y_ion
prec
b2+
b3+
y4+
b5+
y5+
y11++
y7+
y8+
y9+
y10+
b11+
m/z
AKTNADTDGMoVK+XL_L
y2+
b3+
y5+
b5+
y6+
y7+
b7+
y8+
b8+
y9+
y10+
ALLNNSHYYHMoAHGKDFASR+XL_S
B_b_ion
B_y_ion
y18+++
y4+
b14+
ALLNNSHYYHMoAHGKDFASR+XL_L
b13++
y18+++
b16++
y4+
y10+
y12+
